# The Central Role of Learning in Preventing Foot Complications in Persons With Diabetes: A Scoping Review

**DOI:** 10.1111/jocn.17678

**Published:** 2025-02-25

**Authors:** Kristofer Björk, Susanne Andersson, Ulla Hellstrand Tang, Henrik Eriksson

**Affiliations:** ^1^ University West Department of Health Sciences Trollhättan Sweden; ^2^ Research and Development Centre, Primary Health Care, Skaraborg Skövde Sweden; ^3^ Department of Prosthetics and Orthotics Sahlgrenska University Hospital Gothenburg Sweden; ^4^ Department of Orthopaedics, Institution of Clinical Sciences, Sahlgrenska Academy University of Gothenburg Gothenburg Sweden

**Keywords:** diabetic, educational interventions, foot care, learning strategies, patient education, scoping review, self‐efficacy

## Abstract

**Background:**

Despite a variety of literature reviews, there is limited understanding of the learning strategies healthcare professionals use to help patients adopt and maintain effective foot care practices.

**Aim:**

To explore learning processes and educational strategies for persons with diabetes focusing on foot care and examine how different learning processes influence these strategies.

**Method:**

The scoping review followed the methodological framework established by Arksey and O'Malley and refined by Levac et al. Additionally, the PRISMA‐ScR checklist was followed. A literature search was conducted in the PubMed, CINAHL, MEDLINE and Academic Search Premier databases, using specific search terms related to diabetic foot care and learning strategies. The selection process involved screening 906 articles based on inclusion criteria such as relevance to diabetic foot care, learning processes, and educational strategies, and excluded studies that were not written in English. The data were charted and quantitative and qualitative data were synthesised and thematically analysed to identify key learning strategies.

**Results:**

The analysis identified two main themes: learning insights for comprehensive understanding and self‐efficacy, and increased practical knowledge leads to improved footcare. Subthemes included integrative and reflective learning, motivational and collaborative learning, task‐oriented and procedural learning, and feedback and reinforcement‐based learning.

**Conclusion:**

Effective diabetic foot care education should be multifaceted, incorporating deep learning, practical skills, and motivational elements. Early learning plays a central role in this process. Tailoring educational interventions to personal learning styles and providing continuous support can significantly improve patients' foot care outcomes. A framework for understanding the progressive stages of patient learning and self‐management is presented as a starting point. Future research should focus on developing and evaluating educational models that address diverse learning needs, ultimately contributing to better management and prevention of diabetic foot complications.

**Summary**

- What does this paper contribute to the wider global community?
  ○ This is the first review to explore learning processes from the perspective of persons with diabetes, focusing on how they internalise and practice personal knowledge for preventing foot care. This approach shifts the focus from traditional educational methods to understanding the patient's learning journey.
  ○ This review highlights significant gaps in the current understanding of how patients learn about diabetic foot care. It emphasises the need for more research into the learning processes that support effective self‐care practices.

## 1 Introduction

This manuscript uses the term ‘persons with diabetes’, as each person with the disease is unique, which aligns with the principles of person‐centred care (McCormack and McCance 2017). Literature reviews have focused on protocols, guidelines, support, and self‐care models regarding foot care for persons with diabetes (Alshammari et al. 2023; Bus et al. 2024; Goodall et al. 2020; Manickum et al. 2021; Naughten et al. 2023; Paton et al. 2021; Yıldırım Ayaz et al. 2022). These reviews have contributed to our understanding of effective foot care practices, which have provided valuable frameworks for both the multidisciplinary teams that support persons with diabetes and for the patients themselves. However, there has been minimal interest in the learning strategies that patients employ or require to engage in self‐care of the feet. It is crucial to understand how patients learn and internalise information about self‐care because this directly impacts their ability to implement self‐care practices and engage in self‐care practices developed in dialogue with healthcare providers (Hill et al. 2022). Learning is a continuous, lifelong process, as persons constantly acquire new knowledge and skills in response to life's challenges and social contexts (Jarvis et al. 2003). Experiential learning plays a significant role in this process, highlighted by Dewey's ‘learning‐by‐doing’ (1997) and Kolb (2014), which both emphasised that learning is driven by direct experience. Other perspectives, such as Skinner's, see learning as behavioural, with examples including skill development (Skinner 1938), or cognitive, for which reflective learning is a key concept (Piaget 1989). Additionally, socio‐cultural learning, described by Vygotsky and Cole (1978), underscores the importance of social interactions and context. Jarvis and Parker (2005) defined learning as a comprehensive process that engages a person's entire body and mind within a social environment, leading to cognitive, emotional, or practical transformation. Kneck et al. (2011, 2012) argued that learning to manage diabetes is an ongoing process that involves both cognitive and experiential learning, where patients initially rely on external information and gradually develop embodied knowledge. Social interaction and sharing experiences with others play crucial roles in adapting to diabetes, helping patients apply learned strategies to their own lives. Understanding these theoretical perspectives is essential for developing practical educational strategies that can be applied in diabetic foot care. Over time, routines become essential for self‐care, but the ability to adapt when routines are disrupted is key to continued learning and disease management.

Structured education on foot care for patients, their families, and healthcare professionals is a crucial part of preventing diabetes foot ulcers (DFU). The primary goal of such education is to enhance foot self‐care knowledge, motivation, and skills, particularly for persons who are at higher risk of developing DFU. This involves encouraging daily foot checks and promoting proper care practices. To ensure proper self‐care, education should be culturally appropriate, which means that it respects and incorporates the cultural beliefs and practices of the patients, is regularly reinforced, and is tailored to personal needs, which helps achieve understanding and adherence (International Working Group on the Diabetic Foot (IWGDF) 2023). However, it is important to address the challenge of traditional teaching methods, where professionals simply relay facts that patients are expected to understand and apply. Instead, education should be interactive, involving activities that engage patients directly, such as discussions, hands‐on practice, and real‐time feedback, and also collaborative, actively involving patients in the learning process. This approach improves knowledge retention and ensures that patients can translate what they learn into practical self‐care actions (Gabbay et al. 2011). Understanding personal learning styles is crucial for designing educational programs. To further support learning, educators should respect and build on the patient's personal experiences with their condition. The learning process should be collaborative, meaning that healthcare providers and patients work together to identify learning needs and plan educational activities, ensuring that patients are actively involved in their own care (World Health Organization 2023). According to Fleming (2001), there are different types of learning styles. One model for identifying personal learning styles is VARK, where V stands for visual, A for auditory, R for read/write, and K for kinesthetic. Visual learners prefer visual aids like pictures, diagrams, and charts and benefit from colour‐coding and spatial organisation. Aural or auditory learners respond well to verbal information and discussions, remembering stories and analogies, and benefit from talking about what they have learned. Read/write learners prefer written words and benefit from lists, headings, and note‐taking, and find handouts and manuals to be particularly useful (Berglund et al. 2024; Fleming 2001). Kinesthetic learners learn best through hands‐on experiences and real‐life examples, integrating new information with existing knowledge through practical applications (Fleming 2001). To enhance the overall learning experience, it is important to recognise that learning strategies are deliberate actions and techniques that learners employ to improve their understanding, retention, and application of knowledge (Shi 2017). Tailoring education to the patient's learning style is crucial for improving their understanding and self‐care, particularly for persons with diabetes. When educational materials are adapted to different learning styles, such as visual, auditory, read/write, or kinesthetic, patients experience significant improvements in their diabetes knowledge and their health literacy. This personalised approach helps patients understand and manage their condition, which leads to improved outcomes. For instance, patients who receive education in their preferred learning format are more likely to retain critical information, such as recognising symptoms of complications and managing blood sugar levels effectively (Koonce et al. 2015).

The interventions for preventing foot ulcers in persons with diabetes are structured around five key prevention strategies: identifying persons at risk, conducting regular foot inspections, providing education (including psychological support) for patients and caregivers, ensuring the use of suitable footwear, and treating risk factors that could lead to ulcers (IWGDF 2023). While these strategies can form a foundation of foot ulcer prevention, their effectiveness needs to be evaluated in real‐world settings. Alshammari et al. (2023) examined various educational programs designed to improve self‐care of the feet among persons with diabetes. This umbrella review identified a wide range of methods used to implement podiatry education to the intervention groups. The review concluded that patient education programs did not significantly improve the recurrence of ulcers and, in a few cases, the foot education interventions did not lead to a significant reduction in diabetes‐related foot ulcer incidence and amputations. While Alshammari et al. (2023) provided valuable insights into various educational methods, they did not fully explore the learning processes involved how persons with diabetes actually learn and internalise the knowledge and skills needed for adequate foot self‐care. It is crucial to understand these learning processes because they directly impact the sustainability of self‐care practices. Given this context, there is a need to shift the focus from simply evaluating the outcomes of educational interventions to exploring the learning processes that facilitate personalised self‐care in persons with diabetes. By understanding how patients learn and apply their knowledge, we can develop more personalised educational strategies that enhance long‐term self‐care practices and ultimately improve health outcomes for persons with diabetes.

## 2 Aim

The aim of the present study was to explore learning processes and educational strategies for persons with diabetes focusing on foot care and examining how different learning processes influence these strategies.

## 3 Method

The scoping review methodology provided a clear structure for systematically searching the literature, identifying different types of evidence, clarifying concepts and terms, employing methods, and noting patterns and gaps in the literature (Munn et al. 2018). The review was conducted following the methodological framework established by Arksey and O'Malley (2005) and later refined by Levac et al. (2010) for scoping reviews. The framework includes six stages, although the final step consultation is optional and was omitted due to resource and scope constraints; its inclusion could have provided additional insights. The five principal stages of the framework are: identifying the research question; identifying relevant studies; study selection; charting the data; and collating, summarising, and reporting the results. The PRISMA‐ScR (preferred reporting items for systematic reviews and meta‐analyses extension for scoping reviews) Checklist (Tricco et al. 2018) was used to ensure rigour and consistency throughout the scoping review PRISMA extension for scoping reviews (PRISMA‐ScR): checklist and explanation (Data S1).

### 3.1 Identifying the Research Question

When conducting a scoping review of learning processes and educational strategies for persons with diabetes focusing on foot care, the first stage involves formulating the research question. The research question should be broad yet well‐defined to guide the subsequent stages. The framework emphasises the importance of clearly articulating the scope, including defining key concepts, target populations, and desired health outcomes. For this study, the research question has been formulated as: ‘How are learning processes and educational strategies related to foot care described for persons with diabetes? Specifically, how do different learning processes influence these educational strategies?’. To structure the review and ensure clarity, the research question was divided using the PEO (population, exposure and outcome) framework. The review focuses on persons with diabetes who require foot care (P) and learning processes and educational strategies (E) designed to enhance understanding and management of foot care. The outcomes of interest include studies that describe how different learning processes influence the application or discussion of educational strategies in relation to foot care for persons with diabetes (O), allowing for a comprehensive exploration of the existing literature on this topic.

### 3.2 Identifying Relevant Studies

The second stage involves identifying relevant studies, balancing comprehensiveness against practical constraints such as time and resources. The search strategy should be thorough yet feasible to ensure it adequately addresses the research question without being overly exhaustive.

The search of databases was undertaken in March 2024. During the literature search process, the first author also consulted library staff for guidance and support in identifying relevant sources and refining search strategies. The PEO framework was used to structure the review: focusing on persons with diabetes requiring foot care (P), learning processes and educational strategies (E), and descriptions of how different learning processes influence the application or discussion of educational strategies in relation to foot care for persons with diabetes (O). This framework guided the selection of relevant search terms and targeted literature search. The first author performed systematic literature searches in the PubMed, CINAHL, MEDLINE and Academic Search Premier databases without setting any time restrictions. The search terms were selected based on initial manual searches and a pilot search and mapped to CINAHL headings and medical subject headings (MeSH) terms in PubMed. These headings are used in the databases to index articles, but the search terms are also included as free text, guided by the results from the manual and pilot searches, and applied truncation (*) when necessary. Search terms used in CINAHL, MEDLINE and Academic Search Premier were patient*, diabetic foot* and lear* or educational intervention*. The search terms used in PubMed had to be adapted slightly based on the specific database, and then the terms diabetic feet and patient and learning or ‘educational intervention’ were used. All searches were limited to peer‐reviewed literature.

### 3.3 Study Selection

**FIGURE 1:**
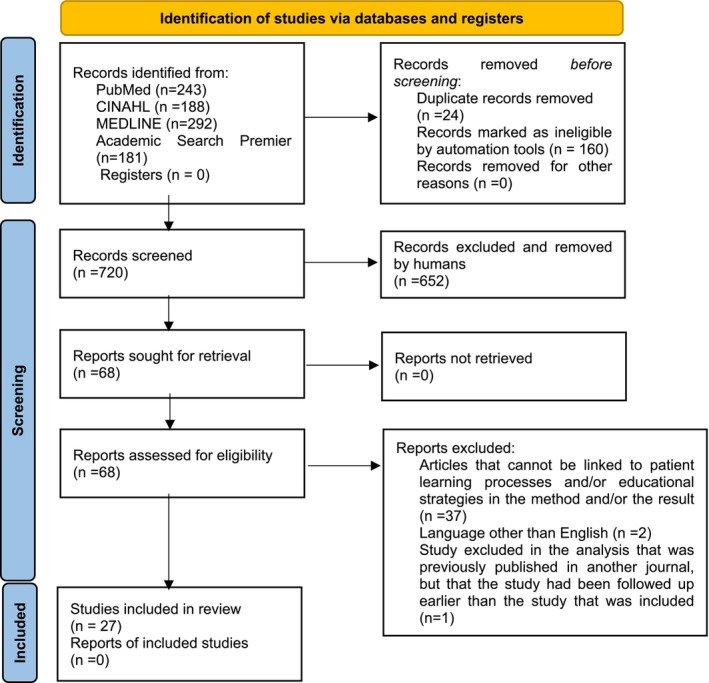
PRISMA 2020 flow diagram for new systematic reviews which included searches of databases and registers only (Page et al. 2021). [Colour figure can be viewed at wileyonlinelibrary.com]

The first and last authors worked closely together during the literature search to ensure that the process followed the method description. In total, 904 articles were searched; after duplicates were excluded, the titles/abstracts of 720 articles were read. From those articles, the method and results of 68 articles were reviewed, which resulted in 27 articles being used for the analysis itself. The following inclusion criteria were applied: (1) the population includes persons with diabetes who require foot care, encompassing both adult and elderly patients; (2) studies must focus on different learning processes and educational strategies related to foot care (exposure); (3) the outcome criteria include studies that describe how different learning processes influence the impact/influence of educational strategies among persons with diabetes. The exclusion criteria were: (1) studies that do not specifically focus on persons with diabetes; (2) studies that include children or adolescents under 18 years of age; (3) studies that do not examine different learning processes or educational strategies related to foot care; and (4) studies that do not describe how different learning processes influence the impact/influence of educational strategies among persons with diabetes. Studies not responding to the purpose of the study were excluded, as were studies written in a language other than English or previously published in another journal, but that the study had been followed up earlier than the study that was included. See Figure 1 for an overview of the process.

### 3.4 Charting the Data

The fourth stage involved charting the data from the selected studies. The process included developing a data mapping form to determine which variables to extract where the author, year of publication, study details, population and learning strategies are presented in Table 1. The numerical summary can be found in Table 1. The charting process was iterative, with the data mapping form being refined as familiarity with the data increased, which ensured comprehensive capture of relevant aspects. The studies in this review show significant variation in study design and data collection periods, ranging from short intervention studies to longer follow‐up studies.

**TABLE 1:** Overview of included studies.

Author year	Type of study	Population	Learning strategies
Adarmouch et al. (2017), Marocco	Quantitative	Patients with diabetes (*n* = 199)	Pre‐post prospective quasi‐experimental. The intervention consisted of a culturally sensitive workshop with facilitated group discussion and narrated video content, focusing on minimal foot care practices. Foot care practices were assessed prior to the session and 1 month later
Annersten Gershater et al. (2011), Sweden	Quantitative	Patients with diabetes and sensory neuropathy, healed foot ulcer below the ankle, and a high risk of developing diabetic foot ulcers (*n* = 131)	Participant‐driven patient education in group sessions was compared to standard information regarding the reduction of ulceration in patients with neuropathy and a previous foot ulcer
Anselmo et al. (2010), Brazil	Quantitative	Patients with diabetes and with risk for foot ulcer (*n* = 60)	Multidisciplinary educational approach, evaluation was carried out using a questionnaire scoring from 0 to 10 (high scores reflect worse practice compliance)
Baccolini et al. (2022), Italy	Quantitative	Patients with diabetes who have foot ulcers or are at high risk of developing foot ulcers (*n* = 253)	Motivational approach through group sessions, conducted by a podiatrist and an expert in psycho‐education. Motivation to self‐care and competence were assessed by specific questionnaires
Corbett (2003), USA	Quantitative	Patients with diabetes (*n* = 40)	Educational intervention about foot care topics to improve foot self‐care. Conducted pre‐ and post‐tests to evaluate educational intervention on diabetic foot care
Costa et al. (2021), Canada	Qualitative constructivist grounded theory	Patients with diabetes (*n* = 30)	Participants described observing and asking questions to learn about ulceration and self‐management tasks during wound‐care clinic appointments and social interactions
Cuevas and Carter (2021), USA	Mixed method	Patients with diabetes (*n* = 10)	The evaluation of the study revealed that adherence to foot care recommendations significantly improved through the implementation of online cognitive training
Fayfman et al. (2020), USA	Mixed method	Patients with diabetes (*n* = 40)	Focus group discussion
Gershater and Wessman (2023), Sweden	Qualitative explorative	Patients with diabetes focusing on maintaining foot health after the healing of a diabetic foot ulcer (*n* = 49)	Participant‐driven group sessions inspired by problem‐based learning
Gravely et al. (2011), USA	Quantitative	Patients with diabetes who had been admitted to the vascular services unit under the care of the vascular surgery physician team (*n* = 23)	The training material used was written and/or via video. In order to be able to assess learning, pre‐ and post‐tests were conducted to evaluate educational intervention on diabetic foot care
Hassan (2017), Jordan	Quantitative	Patients with diabetes (*n* = 225)	Ongoing learning reinforcement (2–3 times weekly) by text messaging followed an informal class on diabetic foot care in a community clinic setting. Foot examinations and pretesting occurred just before patients departed the clinic; the post‐test and a final foot examination occurred 12 weeks later. Pre‐test and post‐test scores for knowledge and practice of foot care were conducted
Heng et al. (2020), Singapore	Quantitative	Patients with diabetes who had an active foot ulcer (*n* = 52)	A collaborative approach to patient education. Conducted pre‐ and post‐tests to evaluate educational intervention on diabetic foot care
Ko et al. (2011), South Korea	Quantitative	Newly registered low‐income adults with diabetes (*n* = 96)	Individually tailored education. Conducted pre‐ and post‐tests to evaluate educational intervention on diabetic foot care
Li et al. (2019), China	Quasi‐experimental	Patients with diabetes and retinopathy (*n* = 80)	Twelve‐week educational intervention on foot self‐care behaviour, including bedside visits, leaflets, and videos during hospitalisation, followed by telephone and home visit follow‐ups post‐discharge. Before and after the intervention, subjects filled out the Diabetic Foot Self‐care Behaviour Scale
Makiling and Smart (2020), United Arab Emirates	Quantitative	Patients with diabetes (*n* = 10)	Educational program, conducted pre‐ and post‐tests to evaluate educational intervention on diabetic foot care
Marchand et al. (2018), France	Quantitative	Patients with diabetes (*n* = 109)	Comparison and evaluation of two educational programs through pre‐ and post‐tests to evaluate educational intervention on diabetic foot care
Mohammad and Khresheh (2018), Saudi Arabia	Quasi‐experimental	Patients with diabetes (*n* = 60)	Educational interventions in the prevention of diabetic foot ulcers through knowledge of the disease and self‐care practices. Conducted pre‐ and post‐tests to evaluate educational intervention on diabetic foot care
Monami et al. (2015), Italy	Quantitative	Patients with diabetes (*n* = 121)	Educational program, pre‐ and post‐tests contended to evaluate educational intervention on diabetic foot care
Moradi et al. (2019), Iran	Interventional quasi‐experimental research design	Patients with diabetes (*n* = 160)	Educational intervention based on short message service (SMS). A three‐section questionnaire completed by a face‐to‐face interview used for data collection before and after the intervention and 3 months after the education
Nemcová and Hlinková (2014), Slovakia	Quantitative	Patients with diabetes (*n* = 100)	Interventional diabetic foot care education with verbal and written patient education material. Participants were assigned to receive either individual or group education. Pre‐ and post‐tests conducted to evaluate educational intervention on diabetic foot care
Pezeshki et al. (2023), Iran	Quasi‐experimental intervention	Patients with diabetes (*n* = 100)	Educational program, pre‐ and post‐tests conducted to evaluate educational intervention on diabetic foot care
Satehi et al. (2021), Iran	Quantitative	Patients with diabetes with diabetic foot complications (*n* = 90)	Teach‐back and multimedia teaching methods versus routine care on the self‐care of patients with diabetic foot ulcers. Self‐care test before and after intervention
Singh et al. (2020), India	Quantitative	Patients with diabetes (*n* = 184)	Educational intervention, pre‐ and post‐tests conducted to evaluate educational intervention on diabetic foot care
Toygar et al. (2022), Turkey	Quasi‐experimental	Patients with diabetes (*n* = 33)	Educational program, pre‐ and post‐tests conducted to evaluate educational intervention on diabetic foot care
Vakilian et al. (2021), Iran	Quantitative	Patients with diabetes and foot ulcer (*n* = 74)	Educational intervention. Before and after the intervention the patients carried out Diabetes Foot Care Self‐Efficacy Scale and Health‐Promoting Lifestyle Profile Questionnaire for diabetic foot care
Vatankhah et al. (2009), Iran	Quantitative	Patients with diabetes (*n* = 148)	Educational program on the knowledge and practice. Evaluation of the participants' knowledge on diabetic foot care before and after the education
Yuncken et al. (2018), Australia	Quantitative	Patients with diabetes (*n* = 24) and their treating podiatrist (*n* = 3)	Baseline data collection included educational topics and delivery methods discussed during the consultation. The Problem Areas in Diabetes Questionnaire and perceived key educational message were collected from each group's perspective at baseline and 6 months afterwards

### 3.5 Collating, Summarising and Reporting the Results

After charting, narrative summaries of the quantitative findings were combined with the qualitative findings and subjected to coding process focus on learning. A thematic analysis departing from personal and social learning theories (as presented in the background) using qualitative content analysis was conducted to identify and categorise broader patterns related to learning within the research field. Additionally, the thematic analysis synthesised information and provided comprehensive understandings of the included studies, which led to the identification of two main themes and four subthemes, as shown in Tables 2, 3, 4, 5, 6. While traditional thematic analysis focuses on the deep exploration of individual data points, our approach focused on the mapping of overall trends. The first and last authors worked together during data extraction and initial analysis, ensuring methodological rigour and accurate representation of data. All authors participated in iterative discussions upon the initial analysis, examining the presentations of the data referring to and forward‐creating the final version of findings. This thematic analysis provided a structured framework for understanding how different learning processes influence educational strategies. No formal quality appraisal of the articles was conducted; this approach aligns with those of Arksey and O'Malley (2005) and Levac et al. (2010), who did not emphasise this step. However, the first author ensured that all included articles were scrutinised for research ethics and that all studies had received ethical approval.

## 4 Results

The results of this study highlight two key themes in the learning processes that are essential for personalised care of patients with diabetes and their feet. First, learning is crucial for developing a comprehensive understanding of diabetes management and enhancing self‐efficacy among patients. This process involves integrative, reflective, motivational, and collaborative learning approaches, which together deepen understanding and improve care outcomes. Second, increased practical knowledge leads to improved foot care, focusing on task‐oriented, procedural learning supported by regular feedback and reinforcement. These combined learning processes are essential for fostering the skills and behaviours necessary to manage diabetes and prevent complications. The results of the thematic analysis are presented in Table 2.

**TABLE 2:** Result of the thematic analysis.

Theme	Subtheme
Learning insights for comprehensive understanding and self‐efficacy	Integrative and reflective learning Motivational and collaborative learning
Increased practical knowledge leads to improved foot care	Task‐oriented and procedural learning Feedback and reinforcement‐based learning

### 4.1 Learning Insights for Comprehensive Understanding and Self‐Efficacy

This theme focuses on methods that promote deep learning and understanding of foot care among patients with diabetes, encouraging critical thinking, self‐efficacy, and sustainable behavioural transformation.

#### 4.1.1 Integrative and Reflective Learning

This subtheme captures learning approaches that provide knowledge, but also encourage participants to reflect on their personal experiences and the broader implications of their condition. These methods integrate cognitive principles, such as problem‐based learning and therapeutic education, to deepen understanding of the disease and help patients grasp what it means to live with and manage diabetes. The goal is to promote a deeper sense of self‐efficacy and empower patients to take control of their foot care through reflective practices. The combination of cognitive tools and practical exercises helps connect theoretical knowledge with real‐life applications. The articles in the integrative and reflective learning subtheme are presented in Table 3.

Marchand et al. (2018) incorporated cognitive psychology principles and innovative learning tools to enhance patient understanding and self‐efficacy, encouraging patients to connect their experiences with disease management and apply their knowledge to real‐life situations. Gershater and Wessman (2023) used problem‐based learning to deepen insights into ulcer prevention, appropriate footwear, and timely help‐seeking, with participants acknowledging the need for more consistent and informed foot care. That study highlighted the challenges of obtaining personalised footwear, the importance of skilled chiropodists, and the crucial role of family support in managing diabetic foot health. Vakilian et al. (2021) used comprehensive education based on Pender's Health Promotion Model (HPM), which addressed understanding obstacles and benefits, managing feelings about foot ulcers, enhancing interpersonal interactions, and designing a care plan. The education was provided through lectures and discussions and was assessed and followed up with phone contact. These results align with the principles of reflective learning by integrating educational techniques that encourage participants to engage actively with the material and reflect on their personal health behaviours. Cuevas and Carter (2021) found that the learning was not specifically focused on foot care, but on improved adherence.

**TABLE 3:** Articles in the integrative and reflective learning subtheme.

Participants	Intervention	Outcome	Notes
10 participants (aged 40–65)	Cognitive training, webinar classes with online computer game training	Adherence to foot care recommendations were improved by online cognitive training	Cuevas and Carter (2021), USA
49 participants (aged 46–78)	Group PBL, learning important strategies to prevent re‐ulceration	Participants recognised the importance of prevention and self‐care for diabetic foot health, including appropriate shoe choices, developed new strategies to manage injuries, and emphasised the need for timely assistance as well as support from family and peer education	Gershater and Wessman (2023), Sweden
109 participants: control group 53 (aged 55–72) and intervention group 56 (aged 55–72)	Group education, therapeutic patient education, workshop learning tools (posters, card games, foot problem photos), cognitive psychology principles to get self‐efficacy and disease control	While both groups started with similar scores, intervention group showed significant improvement in understanding the disease and prevention behaviours from T0 to T1, with overall improvements seen in all 109 patients, particularly those with involved relatives. However, despite the education provided, approximately one in five patients still developed a new ulcer	Marchand et al. (2018), France
74 participants: intervention group 37 (aged 32–84) and control group 37 (aged 28–82)	Individual and group education was delivered through lectures, individual and group discussion, questions‐and‐answers, and an educational booklet	The intervention was effective at improving the lifestyle habits and self‐efficacy of the patients in the intervention group	Vakilian et al. (2021), Iran

#### 4.1.2 Motivational and Collaborative Learning

This subtheme explores the role of social interactions and motivation in learning. It encompasses methods that leverage group dynamics, peer support, and motivational strategies to foster a supportive learning environment. These approaches are designed to encourage patients to share experiences, learn from each other, and maintain a commitment to foot care practices over time. The collaborative aspect enhances learning by creating a sense of community and shared responsibility, which can be particularly beneficial for sustaining behavioural change. These methods also often incorporate motivational elements that help patients overcome barriers to self‐care. The articles in the motivational and collaborative learning subtheme are presented in Table 4.

Mohammad and Khresheh (2018) found that structured, interactive sessions on diabetes care, including foot care, increased patient interest and knowledge through active participation and discussion, aligning with the core principles of motivational learning. Similarly, Annersten Gershater et al. (2011) emphasised the importance of reflective group discussions, where patients explored the origins of foot ulcers together, fostering a deeper understanding and reinforcing their confidence in managing their condition. Fayfman et al. (2020) highlighted the importance of peer learning and social support in maintaining foot care routines. Pezeshki et al. (2023) utilised a comprehensive approach that combined group discussions and motivational tools such as leaflets and weekly messages. This education included lectures, demonstrations and practice of foot care techniques, supported by videos and PowerPoint presentations, as well as question‐and‐answer sessions during the course. Adarmouch et al. (2017) also emphasised interactive group discussions combined with multimedia tools to enhance engagement and learning outcomes. Costa et al. (2021) demonstrated how motivational and collaborative learning processes are integral to helping patients transition from initial awareness to confident self‐management of their condition. That study emphasised the role of social networks, healthcare professionals, and personal determination in promoting a supportive environment where patients can develop and sustain self‐care routines. Nemcová and Hlinková (2014) illustrated the importance of motivational and collaborative learning in diabetic foot care education, showing how structured education, delivered through both personal and group sessions, can enhance patients' knowledge and motivation, ultimately leading to better self‐care and prevention of severe complications. Heng et al.'s (2020) collaborative method is grounded in the principle that patients are the experts regarding their own lives, and their intrinsic motivation is key to achieving lasting changes in health behaviours. By involving patients as active participants in the educational process, the researchers created a dynamic in which clinicians and patients work together to co‐create treatment plans. This collaborative method respects the patient's autonomy and leverages their own knowledge and experiences, fostering a meaningful and personalised learning experience. Baccolini et al. (2022) focused on motivational interviewing to enhance foot care competence and motivation. Their program emphasised prevention, healthy behaviours and overcoming barriers to change, using practical demonstrations and collaborative communication to strengthen patient commitment and empowerment.

**TABLE 4:** Articles in the motivational and collaborative learning subtheme.

Participants	Intervention	Outcome	Notes
199 participants were recruited and 133 completed the second assessment (aged 44–66)	Interactive group discussions, PowerPoint presentation, video and CD‐ROM of video to take home	Average foot care score increased a month after the intervention, with a portion of participants improving their foot care routines, and literacy being associated with a higher likelihood of positive change, while previous education about diabetic foot was linked to a lower likelihood of improvement	Adarmouch et al. (2017), Marocco
131 participants: 61 in the intervention group (aged 37–78) and 70 in the control group (aged 35–79)	Group discussions. Written and oral instructions control group	After 6 months, 42% of patients developed new foot ulcers, with no significant difference between groups, and the small sample size limited the study's conclusions. Five patients had died, and 21 were lost to follow‐up or declined further participation	Annersten Gershater et al. (2011), Sweden
253 participants (aged 46–78): 81 in the intervention group and 172 in the control group	Group information, supported by slides and practical demonstrations. The communicative style of each session was collaborative and aimed at strengthening motivation and commitment to change, in keeping with the principles of motivational interviewing	Experimental psychoeducational program improved patient adherence to follow‐up and significantly reduced the prevalence of foot lesions compared to standard care, which showed stable lesion rates. Additionally, the psychoeducational program resulted in a lower cumulative incidence of new lesions and a longer time before new lesions developed	Baccolini et al. (2022), Italy
30 participants, most of whom (*n* = 17) were > 65 years of age	Asking questions to learn, constructivism ‘learn by others’, and patient empowerment	The study traced the patients' development from discovering their diabetes and relying on social networks for initial learning, through engaging with specialised care and seeking guidance, to ultimately adopting independent self‐management practices and daily routines for diabetic foot care	Costa et al. (2021), Canada
40 participants (aged 25–> 65 years)	Focus group discussion of how to learn	Although participants in both the ulcer and amputation groups had some understanding of and practices for proper foot care, they often learned about it only after complications arose, felt that essential information was lacking earlier in their disease course, and strongly advocated for more educational resources and peer support, favouring in‐person discussions and interactive learning methods, while also showing interest in both negative and positive‐framing approaches to education	Fayfman et al. (2020), USA
52 participants (42 completed the study): 33 in the intervention group (aged 45–66) and 19 in the control group (aged 50–71)	Individual education, (collaborative patient education and professionals active listening)	The collaborative patient education group demonstrated greater knowledge retention and improved self‐care behaviours than the control group	Heng et al. (2020), Singapore
60 participants: 30 in the intervention group (aged 20–49) and 30 in the control group (aged 20–49)	Group education, feedback from the patients through health education and discussion	This study shows a significant relationship between levels of patients' knowledge, practice and level of education	Mohammad and Khresheh (2018), Saudi Arabia
100 participants (aged 55–68)	Group, individual, verbal and written information	Education can increase knowledge, willingness, and motivation to learn and change the behaviour of diabetics. This applies regardless of whether the education is an individual or in a group, as there was no difference in the knowledge test results between these two groups	Nemcová and Hlinková (2014), Slovakia
100 participants: 50 in the intervention group (aged 40–54) and 50 in the control group (aged 40–55)	Group discussion, lecture, demonstration and practice of proper foot care practices, as well as question‐and‐answer sessions. Leaflet and weekly messages	There was an increase in the patients' knowledge and their ability to continue performing foot care activities in the intervention group compared to the control group immediately after the intervention and 3 months later	Pezeshki et al. (2023), Iran

### 4.2 Increased Practical Knowledge Leads to Improved Foot care

This theme addresses learning methods that are more focused on basic knowledge and practical skills aimed at changing specific behaviours related to foot care.

#### 4.2.1 Task‐Oriented and Procedural Learning

This subtheme focuses on the step‐by‐step learning of essential foot care tasks, emphasising practical skills and procedural knowledge. These methods are designed to ensure that patients understand and can competently perform daily foot care routines, such as washing, trimming toenails, and choosing appropriate footwear. The goal is to instil these tasks as habitual behaviours that can be easily integrated into the patient's life. This type of learning often involves demonstrations, hands‐on practice, and clear instructions to ensure that patients can replicate the procedures independently at home. Patient learning in diabetic foot care is personalised when it combines practical instruction with reinforcement and support. The articles in the task‐oriented and procedural learning subtheme are presented in Table 5. Toygar et al. (2022) emphasised hands‐on, immediate learning through focused sessions on essential foot care tasks, such as foot assessment and nail care. Vatankhah et al. (2009) enhanced this approach with a comprehensive educational program that includes face‐to‐face interactions and a booklet for ongoing reference so that patients can revisit and reinforce their learning. Anselmo et al. (2010) advanced the idea further by offering a structured, multi‐step program that integrates personal consultations, personalised nursing sessions, and group education, involving family members and caregivers for added support. This multi‐layered approach, which combines direct practice, repeated reinforcement, and social support, ensures that patients not only learn, but also consistently apply proper foot care practices in their lives. Ko et al. (2011) and Singh et al. (2020) provided tutoring and education on assessing the severity of the disease and understanding both emerging and expected complications. Training patients regarding foot care includes the techniques and methodology of foot care. Makiling and Smart (2020) argued that lectures accompanied by clinical demonstration are the preferred mode of teaching in a clinical setting given adult learning needs as identified by the patients themselves. Preventive diabetic foot self‐care education was conducted through a lecture, visual aids, and a return demonstration.

Monami et al. (2015) and Corbett (2003) focused on providing patients with essential, task‐oriented education on diabetic foot care, emphasising the practical steps necessary to prevent complications such as ulcers. These studies share a common approach in terms of offering straightforward, actionable guidance to patients, but they do so through slightly different methods and levels of detail. Both studies emphasised the importance of straightforward, practical education to empower patients to manage their foot self‐care. However, while Monami et al. (2015) took a more structured and interactive group‐based approach, Corbett (2003) offered a personalised, one‐on‐one educational experience. Despite these differences, both approaches share the goal of providing patients with the tools and knowledge necessary to prevent diabetic foot complications through consistent and proper foot care practices. Yuncken et al. (2018) underscored the challenges of ensuring that patients learn, retain, and apply specific procedural tasks over time. That study's focus on foot inspections and wound‐care procedures directly aligns with this type of learning. However, it also revealed that patient retention of these tasks is often limited, which highlights the importance of personalised teaching strategies that reinforce learning and promote long‐term understanding.

**TABLE 5:** Articles in the task‐oriented and procedural learning subtheme.

Participants	Intervention	Outcome	Notes
60 participants (aged 32–82)	Individual visit from a nursing professional directed towards general preventive care and review of the medical prescriptions. Group education, patient, family and caregivers	While most patients adhered to proper foot care practices such as moisturising, washing, and inspecting shoes, and avoided walking barefoot or using abrasive objects, only a small percentage consistently wore the provided footwear	Anselmo et al. (2010), Brazil
40 participants (aged 38–91): 20 in the intervention group (19 completed the study); and 20 in the control group (16 completed the study)	Individual education, focused on factors affecting foot care	Educational intervention improved patients' knowledge, confidence and reported foot care behaviours	Corbett (2003), USA
96 participants (aged 25–86)	Individual tailored education	Tailored education significantly improved patients' knowledge of diabetes and self‐management, highlighting the importance of regular, individualised education from nurses for maintaining effective self‐management in low‐income adult diabetic patients	Ko et al. (2011), South Korea
10 participants (aged 40–70)	Group discussions, a lecture, visual aids and a return demonstration	The most prominent educational need was correct toenail cutting, which remained a low‐scoring area despite the intervention, while understanding of the dangers of walking barefoot improved from 60% to 100% post‐education, and participants recognised the importance of seeking professional care for corns and calluses	Makiling and Smart (2020), United Arab Emirates
121 participants (120 completed the study): 60 in the intervention group (aged 63‐81 ) and 60 in the control group (aged 58‐81)	Group education, face‐to‐face lesson on risk factors for foot ulcers, and an interactive session with practical exercises. Control group leaflet written recommendation	The study was concluded earlier than planned due to a highly significant difference in outcomes between the two treatment groups. During the six‐month follow‐up, ulcers developed in six patients, all of whom were in the control group	Monami et al. (2015), Italy
184 participants (aged 21–78)	Individual education, tutoring and education on how to assess the severity of the disease and understand the emerging and expected complications	The study evaluated the absolute and relative learning gain regarding the awareness and knowledge of foot care among diabetic individuals	Singh et al. (2020), India
33 participants (aged 38–72)	Individual education, one session and assessment of the foot	The intervention had a positive impact on the patients' self‐efficacy in foot care, their understanding of diabetic foot care, their overall health status and their quality of life, irrespective of their sociodemographic and disease‐related characteristics	Toygar et al. (2022), Turkey
148 participants (aged 52–59)	Single education and booklet (written information)	The total knowledge score of the studied population was relatively low before the education was provided. However, this value increased after a period of primary assessment and education	Vatankhah et al. (2009), Iran
24 participants (aged 50–72)	Verbal education	Patients could not recall the main message from their meetings after 6 months	Yuncken et al. (2018), Australia

#### 4.2.2 Feedback and Reinforcement‐Based Learning

This subtheme explores the use of feedback mechanisms and reinforcement strategies to solidify learning and promote consistent self‐care of the foot behaviours. By incorporating tools like multimedia, teach‐back methods, and regular follow‐ups, these approaches aim to reinforce the knowledge and skills acquired during education sessions. Feedback allows patients to correct mistakes and refine their practices, while reinforcement helps to maintain these behaviours over the long term. The use of follow‐up sessions, either in person or through digital means like SMS, ensures that patients remain engaged and continue to apply what they have learned, reducing the risk of them relapsing into poor foot care habits. The articles in the feedback and reinforcement‐based learning subtheme are presented in Table 6.

Satehi et al. (2021) demonstrated how teach‐back and multimedia interventions can reinforce patient self‐care, while Hassan (2017) and Moradi et al. (2019) utilised daily follow‐up via SMS to maintain patient engagement in foot care. Li et al. (2019) focused on learning during hospitalisation through weekly bedside visits, where patients were provided with educational materials like leaflets, DVDs, and WeChat videos. The intervention continued after discharge with weekly phone follow‐ups and home visits at 6 and 12 weeks to ensure that patients engaged with the materials consistently. In Hassan's (2017) study, learning through informal small‐group discussions was supplemented by personalised instruction upon request. Patients received a pamphlet summarising the key educational content and were sent regular mobile phone text messages to reinforce daily foot care practices. Gravely et al. (2011) compared written, video‐based, and combined educational materials for diabetic patients during hospitalisation. Results showed that the video‐based method improved patient knowledge and compliance.

The two main themes identified in the results provide insights to aid in comprehensive understanding and self‐efficacy, and increased practical knowledge leads to improved footcare. Integrative and reflective learning, together with motivational and collaborative methods, promote deeper understanding and improve care outcomes. Practical learning, with a focus on task‐oriented and procedural learning, is supported by regular feedback and reinforcement. These combined learning processes are essential for developing the skills and behaviors necessary to manage diabetes and prevent complications.

**TABLE 6:** Articles in the feedback and reinforcement‐based learning subtheme.

Participants	Intervention	Outcome	Notes
23 participants (aged 31–87).	Individual education. (written information, video, and both written and the video education material)	Participants who used the video method showed greater improvements in scores from pre‐test to post‐test than those who received written educational materials, with most video users watching the video multiple times during their hospital stay, while only half of the written materials recipients read them	Gravely et al. (2011), USA
225 participants (aged < 20–> 61)	Group discussion, pamphlet (written information) and short SMS	The study found that while 76% of participants initially had poor foot care practices, this number dropped significantly to < 1% after 12 weeks, alongside gains in knowledge and overall understanding of the disease	Hassan (2017), Jordan
80 participants (aged 53–77 years)	Individual education leaflet, DVD or WeChat video at the hospital and subsequently at home	The study found significant improvements in foot self‐care behaviours among patients with diabetic retinopathy and visual disability, as well as their primary caregivers, following a 12‐week educational intervention	Li et al. (2019), China
160 participants: 80 in the intervention group (aged 38–58) and 80 in the control group (aged 39–55)	Individual education and daily follow‐up by SMS	After the training in the intervention group, patients' awareness of foot care in diabetes improved significantly, with the SMS‐based educational intervention enhancing foot care awareness, behaviours, and metabolic control	Moradi et al. (2019), Iran
90 participants: 30 in the teach‐back group (aged 44–67), 30 in the multimedia group (aged 40–73), and 30 in the control group (aged 44–68)	Individual education (orally with feedback of what they understood), or multimedia (videos via CD, DVD and mobile device files) Control group that offered routine care, including educational recommendations	Both teach‐back and multimedia interventions had a positive impact on patient self‐care compared to the control group	Satehi et al. (2021), Iran

## 5 Discussion

**FIGURE 2:**
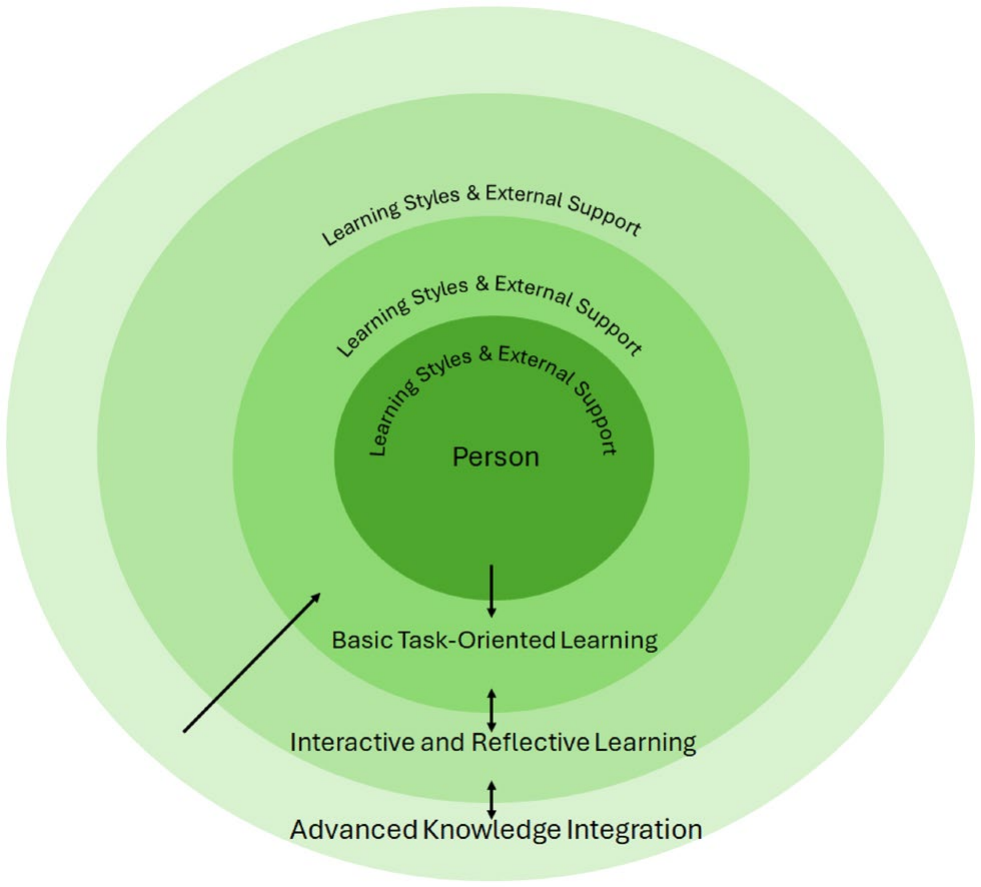
Circular learning strategies and approaches for diabetic foot care. The learning process in the circular model is dynamic, illustrating movement between the stages rather than a static progression. In the innermost circle, acceptance is fundamental, where the person begins by coming to terms with his or her diagnosis through personal reflection and bodily cues. This acceptance is not simply acknowledgement, but an ongoing process in which the person learns to understand their new reality, both cognitively and emotionally. As the person moves outward, he or she engages with more practical learning tasks, followed by deeper interaction and reflection, all while continuously revisiting previous stages to reinforce understanding and adapt to new challenges. The arrows highlight this continuous, iterative movement between circles, indicating that learning is never fully complete, but evolves with the person's growing experience and the support that he or she receives. [Colour figure can be viewed at wileyonlinelibrary.com]

The aim of this study was to explore learning processes and educational strategies for persons with diabetes, with a focus on self‐care of the feet. Accordingly, it is essential to consider which learning strategies have been presented in the literature and how these can be understood within a broader context of lifelong learning. Figure 2 summarises the different learning strategies presented in the results.

Figure 2 was developed in the context of person‐centred learning, inspired by McCormack and McCance's (2017) person‐centred practice framework. It reflects a shift towards understanding learning from the person's perspective, emphasising how persons engage with and progress through their unique learning journey. Figure 2 contains four concentric circles, each of which represents a different stage in the person's learning process.

### 5.1 The Role of Self‐Efficacy

In the innermost circle, representing the first stage, self‐efficacy plays a crucial role, particularly when confronting the reality of the diagnosis. At this stage, the person must begin by acquiring knowledge, but also by understanding and adapting to their condition through personal experiences, bodily signals, and emotional responses, which are essential for engaging in self‐care. Bandura's (1997) theory of self‐efficacy supports this by asserting that individuals' belief in their own abilities influences their willingness to take on challenges and their persistence when facing setbacks. Berglund and Källerwald (2012) noted that the will to maintain independence drives learning, and this is a crucial motivator. This desire is especially important for persons with diabetes because it fuels their commitment to self‐care and adapting to long‐term health challenges. Self‐efficacy is a key factor in diabetes management, particularly in foot care. Qin et al. (2020) and Ahmad Sharoni et al. (2018) found that higher self‐efficacy significantly improves foot care behaviours in persons with diabetes, supporting the integration of self‐efficacy into diabetes education. Our findings reinforce the conclusions of those studies by showing that increased self‐efficacy leads to better foot care and a reduced risk of complications. Furthermore, a high level of self‐efficacy enables the person to achieve long‐term behavioural changes and progress to higher levels of learning and self‐directed care (Bandura 1997). The third circle shows the transition from mastering basic skills to actively engaging with others, such as healthcare professionals and peers, to deepen their understanding. This interaction is crucial because it allows persons to ask questions, receive feedback, and gain a clearer idea of how to manage their self‐care of the feet. Through this reflective learning process, persons are better prepared for the more complex tasks found in the outermost circle. Kjellsdotter et al. (2020) and Berglund et al. (2024) argued that group reflection plays a crucial role in enhancing patient learning by allowing persons with diabetes to share experiences and receive emotional support. This collective reflection fosters deeper self‐awareness and helps patients critically evaluate their self‐care routines, aligning with the reflective learning stage in Figure 2. By engaging in reflective learning, persons with diabetes begin to think critically about their daily routines and how these impact their long‐term health outcomes, which is essential for progressing to more advanced stages of self‐care. When persons with diabetes reach the final stage and develop strong self‐efficacy, they can begin to internalise skills and knowledge that previously required external guidance. This leads to a deeper understanding of the connection between their actions and outcomes, as well as greater confidence in their own abilities. According to Bandura (1997), self‐efficacy is central to setting and achieving goals, which is essential for the person's continued development and independence. This final circle reflects advanced knowledge integration, where persons with diabetes understand the complex relationships between daily routines and long‐term health outcomes. Thus, the theory reinforces the importance of self‐efficacy as a key component in the person's care process (Bandura 1997). This concept of self‐efficacy seamlessly integrates with Figure 2's emphasis on deep learning, which involves moving beyond superficial understanding and memorization to develop a more comprehensive and integrated grasp of a subject. This is most prominent in the outermost circle of Figure 2, where persons with diabetes are expected to integrate advanced knowledge and understand the complex relationships between their daily routines and long‐term health outcomes. This advanced stage of learning involves metacognition, where persons become aware of their own learning processes and can adjust their strategies accordingly. By reaching this advanced stage of learning, persons with diabetes not only demonstrate a high level of self‐efficacy, but also engage in deep learning, which is crucial for sustaining long‐term health behaviours (Winne and Azevedo 2014).

### 5.2 Increased Practical Knowledge Leads to Improved Foot Care

This concept focuses on methods that emphasise the acquisition of fundamental knowledge and the development of practical skills aimed directly at altering specific behaviours related to foot care. In Figure 2, this is particularly evident in the second circle, labelled ‘Basic task‐oriented learning’. Here, the emphasis is on task‐oriented learning, where change is driven by repetition and reinforcement (Watson and Kimble 1998). Persons with diabetes engage in essential self‐care activities, such as washing feet or trimming nails, and receive immediate feedback. This hands‐on practice helps to solidify basic habits that are necessary for foot care. The practical nature of these tasks ensures that the learning is not only retained, but also directly applicable to the person's daily routines, thereby facilitating a smoother transition to sustainable behavioural change. This theme highlights the importance of grounding behavioural change in concrete, practical learning experiences that equip persons with diabetes with the skills they need to maintain their foot health. Paton et al. (2021) highlighted the importance of combining foot care education with practical skills training and supportive measures, such as providing foot care equipment and daily reminders, to improve patients' foot care behaviours. This aligns with the findings from the present study, which emphasise that practical learning methods and regular feedback are crucial for promoting behavioural changes in persons with diabetes. These results are consistent with the insights presented in Figure 2 and the overall findings of this study. Similarly, Kjellsdotter et al. (2020) emphasised that group learning is most effective when participants possess a foundation of basic theoretical knowledge about their condition and nutrition. While lectures can provide these essential building blocks, it is through group interaction and reflection that persons with diabetes are able to ask meaningful, specific questions. This reinforces the idea that persons with diabetes must first acquire a solid grasp of basic knowledge before they can fully engage in deeper learning experiences that can enhance their understanding and improve self‐management skills.

#### 5.2.1 Importance of Personal Learning Styles

The results focus on a variety of educational approaches, such as integrative and reflective learning, as well as practical and motivation‐based education, to enhance understanding and behavioural changes in persons with diabetes. These strategies are designed to promote deep learning and self‐efficacy, but do not specifically address how the education is tailored to personal learning styles. The lack of discussion about learning styles means that there is no exploration of how different pedagogical methods can cater to the diverse learning preferences. This argument is supported by the IWGDF (2023), which emphasised that education should be tailored to personal needs in order to achieve understanding and adherence. According to Fleming (2001), persons have one or more distinct learning styles, which mean that instruction must be tailored to each person's specific needs. This is crucial for supporting the person's ability to progress to the next stage in the circular learning for diabetic foot care. Each stage in Figure 2—from acceptance of the condition, through basic task‐oriented learning, to interactive and reflective learning, and finally advanced knowledge integration—requires that learning be presented in a manner that best aligns with the person's personal learning style. Adapting the instruction can ensure that persons with diabetes receive the information they need in a way that suits them best. Additionally, some persons are aware of their own learning style(s) and can actively seek out information, enabling them to move more effectively through Figure 2's various circles and ultimately reach the outermost circle, where they integrate and apply complex knowledge in their self‐care. For others, progression through the stages in Figure 2 may require more additional support. Vygotsky and Cole's (1978) theory of the zone of proximal development (ZPD) explains how persons can progress through the circular learning in Figure 2 for diabetic foot care with support from healthcare providers and/or social networks. At each stage, from acceptance of the condition to advanced knowledge integration, the ZPD serves as a framework to identify what the person can learn with the right support. This support is essential as a person with diabetes progresses through the circles shown in Figure 2. Healthcare providers and/or social support helps the person overcome obstacles by tailoring the instruction to the person's current level of knowledge and learning style, enabling progression through the circles. The ZPD emphasises the social nature of learning and the importance of continuous support to achieve increased independence and self‐care. Throughout the process, the ZPD helps ensure that the person successfully integrates and applies new knowledge in their self‐care.

## 6 Conclusion

This scoping review has highlighted the critical role of learning strategies for the person in the prevention and management of diabetic foot complications. The findings underscore the central role of early learnings in preventing diabetic foot complications. For persons with diabetes, it is important to start, at an early stage, such as integrative and reflective learning, which foster a comprehensive understanding and self‐efficacy. Additionally, practical learning methods, including task‐oriented and procedural learning, are essential for instilling self‐care of foot care behaviours.

The review reveals that while various educational interventions have been implemented, there is a significant gap in understanding the underlying learning processes. Most studies have focused on the outcomes of educational programs rather than the mechanisms through which persons with diabetes internalise and apply the knowledge. This gap suggests a need for future research to explore the learning processes in greater depth, particularly how different pedagogical methods can cater to diverse learning styles.

Moreover, the integration of motivational and collaborative learning strategies has shown promise in enhancing person engagement and adherence to foot care practices. These strategies leverage social interactions and peer support to create a supportive learning environment, which is crucial for sustaining long‐term behavioural change.

The circular learning shown in Figure 2 illustrates the varied and non‐linear process of person learning and self‐management in diabetic foot care. Effective learning involves not only acquiring knowledge, but also understanding and accepting one's condition through personal experiences, bodily cues, and emotions. The process is personalised, with persons with diabetes following different paths and timelines, and sometimes diverging from professional advice yet still achieving self‐care. Healthcare professionals play a key role in offering education, but also flexible, continuous support, adjusting to each person's unique needs and learning pace. Tailored interventions are crucial at each stage, emphasising the importance of professional sensitivity throughout the person's journey.

In conclusion, diabetic foot care education should be multifaceted, incorporating deep learning, practical skills, and motivational elements. By tailoring educational interventions to personal learning styles and providing continuous support, healthcare providers can significantly improve patient outcomes. The results of this study highlight various educational approaches to enhance understanding and the need for behavioural changes in persons with diabetes, focusing on integrative, reflective, motivational, and practical learning. To implement the circular learning model, nurses and educators can use several strategies. The first is integrative and reflective learning through group discussions and problem‐based learning, sessions where persons share experiences and reflect on their foot care practices. The second is motivational and collaborative learning via peer support groups and motivational interviewing to enhance self‐efficacy and maintain commitment to foot care routines. The third is task‐oriented and procedural learning, with hands‐on workshops that include demonstrations and practice sessions to teach essential foot care techniques. The final strategy is feedback and reinforcement‐based learning using multimedia tools and regular follow‐ups, such as instructional videos and digital messenger reminders, to reinforce learning and ensure adherence to foot care practices. When they are tailored to personal learning styles and needs, these strategies can utilise the circular learning model to improve foot care education for persons with diabetes. Future research should focus on developing and evaluating educational models that address these diverse learning needs, ultimately contributing to better management and prevention of diabetic foot complications.

## 7 Limitation

No formal protocols were registered in this scoping review. This decision was made to allow for a more flexible and iterative approach, which aligns with the methodological framework established by Arksey and O'Malley (2005) and refined by Levac et al. (2010). By not strictly adhering to a pre‐registered protocol, we were able to adapt our search strategy and selection process based on insights gained during the course of the study. This approach has allowed for a more dynamic and responsive research process, which is particularly important in a scoping review that aims to map a broad research area. However, not having a formal protocol could limit replicability or introduce some subjective bias in selecting studies. A lead user patient, who represents persons living with diabetes and possesses extensive expertise regarding their own and others' conditions, actively participated in the review of the research question and the study's results.

The searches in CINAHL, MEDLINE, and Academic Search Premier via EBSCOhost were conducted using the same keywords, while MeSH terms were specifically used in PubMed to increase precision. MEDLINE was searched through both EBSCOhost and PubMed to provide different perspectives on the same database, potentially increasing the chances of finding relevant articles. Duplicates were automatically removed within the EBSCOhost searches, but the use of Academic Search Premier, a multidisciplinary database, increased the number of results and could provide relevant articles that were not found in the other databases. A significant limitation was the initial use of the search term ‘lear*’ to capture variations of the word ‘learning’. This term retrieved many irrelevant articles focused on topics such as ‘machine learning’ and ‘deep learning’, which are artificial intelligence methods unrelated to the educational context intended for this review. The search term ‘education’ was initially used, but this term was too broad and resulted in an overwhelming number of hits, which also complicated the identification of relevant studies. To narrow the scope, the search term was further refined to ‘educational intervention’. These limitations in search terms may have restricted the search and potentially excluded relevant studies. Nevertheless, the scope of the literature obtained was considered relevant and sufficient to conduct a thorough analysis. The review did not impose restrictions based on language or publication date, apart from excluding studies written in a language other than English. This approach aimed to be as inclusive as possible, but it may have introduced some bias by excluding non‐English studies that could have been relevant.

It is important to acknowledge certain limitations in the included studies and the review process that may affect how the results are interpreted. Many of the included studies had small sample sizes, which can limit the transferability of the results. Additionally, several studies had short follow‐up periods, making it difficult to assess the long‐term impact of the educational interventions. Cultural factors may have also influenced the learning processes, as educational strategies that are successful in one cultural context may not be as successful in another. These limitations should be considered when interpreting the results and drawing conclusions about the impact of the various educational strategies. Despite these limitations, this study has several positive aspects worth highlighting. The included studies offer a rich diversity of methods and perspectives, providing a comprehensive understanding of the factors influencing learning and educational strategies in diabetic foot care. This diversity contributes to deeper insights into how different educational strategies can be tailored to meet the personal needs of persons with diabetes.

The heterogeneity among the included studies presents both challenges and opportunities for synthesising the findings. Differences in study design, sample sizes, participant characteristics, and intervention details make it difficult to directly compare the results across studies, which limits the ability to draw broad general conclusions. This heterogeneity contributes to a rich understanding of learning processes and educational strategies for persons with diabetes, but also makes it challenging to compare and synthesise the results. Each study provides unique insights that are context‐specific and may not be universally applicable, further complicating transferability. The variability in results and methods means that identifying common trends or patterns can be challenging, which makes it harder to develop universal guidelines or recommendations based on the aggregated results. At the same time, the various methods offer a comprehensive understanding of the factors influencing learning and educational strategies in diabetic foot care. This diversity of methods and results provides a richer picture of how various educational strategies can impact the learning and self‐management of persons with diabetes.

No formal quality assessment of the included studies was conducted. This is in line with the recommendations from Arksey and O'Malley (2005) and Levac et al. (2010), who emphasised that scoping reviews aim to provide an overview of existing research rather than assess the quality of individual studies. By not conducting a critical appraisal, we were able to include a broader range of studies, providing a more comprehensive picture of the research field. This approach is particularly useful when exploring new or complex research areas where it is important to include various types of evidence and perspectives. However, this means that the findings may include studies with different levels of methodological rigour, which could affect the overall conclusions of the review.

Finally, it is worth acknowledging that publication bias might influence the results, as studies with significant findings are more likely to be published. This could lead to an overrepresentation of positive outcomes in the reviewed literature.

## Author Contributions

**Kristofer Björk:** conceptualization, methodology, formal analysis, writing – original draft. **Susanne Andersson:** conceptualization, writing – review and editing. **Ulla Hellstrand Tang:** conceptualization, writing – review and editing. **Henrik Eriksson:** conceptualization, methodology, formal analysis, writing – review and editing.

## Conflicts of Interest

The authors declare no conflicts of interest.

## Supporting information


Data S1.

## Data Availability

This scoping review is based solely on secondary data extracted from previously published primary studies. No new data were created or collected for this study. All data analyzed during this review are publicly available within the included studies, which have been retrieved through a systematic search of the literature. The datasets used are referenced in the manuscript, and access to the original data can be obtained from the respective publications.
